# Shortest path counting in probabilistic biological networks

**DOI:** 10.1186/s12859-018-2480-z

**Published:** 2018-12-04

**Authors:** Yuanfang Ren, Ahmet Ay, Tamer Kahveci

**Affiliations:** 1 Department of Computer and Information Science and Engineering, University of Florida, Gainesville, 32611 FL USA; 2 Departments of Biology and Mathematics, Colgate University, Hamilton, 13346 NY USA

**Keywords:** Shortest path, Probabilistic networks, Edge betweenness, Community detection

## Abstract

**Background:**

Biological regulatory networks, representing the interactions between genes and their products, control almost every biological activity in the cell. Shortest path search is critical to apprehend the structure of these networks, and to detect their key components. Counting the number of shortest paths between pairs of genes in biological networks is a polynomial time problem. The fact that biological interactions are uncertain events however drastically complicates the problem, as it makes the topology of a given network uncertain.

**Results:**

In this paper, we develop a novel method to count the number of shortest paths between two nodes in probabilistic networks. Unlike earlier approaches, which uses the shortest path counting methods that are specifically designed for deterministic networks, our method builds a new mathematical model to express and compute the number of shortest paths. We prove the correctness of this model.

**Conclusions:**

We compare our novel method to three existing shortest path counting methods on synthetic and real gene regulatory networks. Our experiments demonstrate that our method is scalable, and it outperforms the existing methods in accuracy. Application of our shortest path counting method to detect communities in probabilistic networks shows that our method successfully finds communities in probabilistic networks. Moreover, our experiments on cell cycle pathway among different cancer types exhibit that our method helps in uncovering key functional characteristics of biological networks.

## Background

Biological molecules such as proteins and metabolites work together to deliver specific functions inside the cell. Through their cooperative interactions, these molecules carry out key functions, such as transcriptional regulation and signal transduction [1]. Molecular interactions are often modeled as biological networks (graphs), where nodes represent molecules and edges describe the interactions between them [2]. Analyses of biological networks have provided deep insights into understanding biological systems at the molecular level [3–7], such as discovering signaling pathways [8], predicting protein functions [9], and identifying relationships between genes and disease phenotypes [10, 11].

Biological networks share structural properties that are often informative to discover the key components of the biological systems and their functional roles. One such characteristic property is the set of ‘shortest paths’ connecting two given molecules on the network. This measure is often used as a proxy to functional distance between two molecules. Average of shortest paths over all node pairs (mean path length) has been used as a measure of network’s navigability [2]. Shortest path search has been utilized to find functional clusters in biological systems, and to identify core pathways in glioblastoma and genetic determinants of longevity [12–14]. Edge betweenness centrality and network modularity are two closely related network characteristics to shortest path. Edge betweenness centrality, a measure of the number of shortest paths that go through each edge, describes the essentiality of the underlying gene-to-gene interactions, and helps us to discover the bottlenecks in the biological systems [15]. Finding the centrality scores of edges is thus crucial to understand how the biological networks operate, and how they could be fixed or manipulated. Biological networks often contain substructures, called *communities* (also referred as modules or clusters) [2]. One common way to find the communities in networks is to remove the edges with the highest betweenness value iteratively [5]. Nodes in a community often share common properties such as being functionally related [16, 17], thus identifying the community structure is crucial to discover functions of genes and to decipher mechanisms of biological systems.

Counting the number of shortest paths between two genes in a network is a polynomial time problem. However, the fact that biological interactions are inherently stochastic events dramatically complicates this problem since an interaction happens with some probability. This uncertainty follows from the fact that key biological processes governing these interactions, like DNA replication, gene transcription, and epigenetic mutations, are themselves inherently uncertain events. For example, DNA replication can start at various chromosome locations with different probabilities [18]. In addition, other epigenetic factors can alter the expression level of genes, which in turn affect the likelihood of interactions between molecules.

**Fig. 1:**
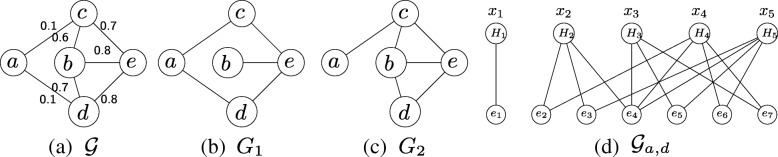
A probabilistic network $\mathcal {G}$ (**a**), its two possible deterministic network topologies *G*_1_ and *G*_2_ (**b,c**) and a sample bipartite graph between nodes *a* and *d* (**d**) are shown. The bipartite graph $\mathcal {G}_{a,d}$ models the dependency between paths connecting nodes *a* and *d* in the probabilistic graph shown in (**a**). *H*_1_, *H*_2_, *H*_3_, *H*_4_ and *H*_5_ are the simple paths between *a* and *d*. Collectively these paths yield seven edges, which are {*e*_1_=(*a*,*d*),*e*_2_=(*b*,*c*), *e*_3_=(*b*,*d*),*e*_4_=(*a*,*c*),*e*_5_=(*c*,*e*),*e*_6_=(*b*,*e*),*e*_7_=(*e*,*d*)}

Existing studies often model the uncertainty of biological interactions using probabilistic networks. Briefly, each edge in the probabilistic network is associated with a probability value showing the confidence in corresponding interaction’s presence. Several large databases, MINT [19] and STRING [20], for instance, already provide interaction confidence values. If a biological network contains at least one uncertain interaction, we call it a *probabilistic network*. Otherwise, it is a *deterministic network*. Figure 1a depicts a hypothetical probabilistic network that has five proteins and seven interactions. An important observation is that a probabilistic network is actually a summary of all possible deterministic networks that are determined by the subset of interactions that takes place. This means that a probabilistic network represented as a graph with |*E*| edges will in fact describe the 2^|*E*|^ deterministic networks. Figures 1b and c present two possible deterministic networks of the probabilistic network in Fig. 1a among 128 (i.e., 2^7^) alternatives. In recent studies, graph polynomials which have a wide range of applications in chemistry, physics and biology [21–25], have also been used to model the probabilistic network [26–28].

Analysis of shortest paths in probabilistic networks received some attention previously [29–31]. However, these studies mainly focused on the problem of finding shortest paths in graphs whose branches are weighted with random lengths. Here, we consider the problem of counting the number of shortest paths between two nodes whose edges are weighted by their interaction probability. This problem has been solved on deterministic network topologies previously [32, 33]. However, the exponential growth of the number of deterministic instances resulting from a probabilistic network makes it infeasible to directly apply existing solutions to probabilistic networks. There are several ways to deal with probabilistic networks. Most of these approaches transform the probabilistic networks to deterministic networks using different mechanisms, such as totally ignoring the interaction probabilities [34, 35], generating random binary networks based on probability [36], or considering only interactions above a given threshold [37]. All these approaches lose some information regarding the topology of the original network. They either over- or under-represent the rare events. *As a result, a scalable method which takes the edge probabilities in probabilistic networks into account is urgently needed.* The fact that some network measures (e.g., betweenness centrality and network modularity) being calculated based on shortest path search algorithm makes a solution to this problem relevant to a wide set of biological questions.

**Our Contributions.** In this paper, we develop a novel method to count the shortest paths between a pair of nodes in probabilistic networks. The key challenge arises from the fact that since the topology of a network is uncertain, existence of paths are also uncertain. Furthermore, this uncertainty is governed through complex dependencies among paths through their shared edges. To capture such dependency between multiple paths, we build a novel polynomial model. Our model first builds a bipartite graph to describe the dependency among multiple paths. It then generates a special class of polynomials, called the *x-polynomial* to express this dependency. We prove that our model accurately counts the distribution of the number of shortest paths. Our experimental results on synthetic and real cancer datasets demonstrate that our algorithm successfully counts the number of shortest paths, while existing methods yield errors ranging from 12% to orders of magnitude depending on the network characteristics. We show that our method can be used to detect essential genes in a network. As a significant application of our novel shortest path counting method, we use it to identify communities in a given probabilistic network. We analyze the community structure in cell cycle pathway among different cancer types. Our results suggest that our method can help in uncovering key functional characteristics of the genes participating in those networks.

We organize the rest of the paper as follows. We present our method in the “Methods” section. We discuss our experimental results in the “Results” section and conclude in the “Discussion and Conclusion” section.

## Methods

In this section, we discuss our shortest path counting method in detail. We present the details on how we adapt this method to compute edge betweenness and to identify communities in networks in “Community detection problem” section.

### Preliminaries

First, we present basic notations needed to define the problem considered in this paper. We denote the set of nodes and the set of interactions among those nodes of a deterministic network with *V* and *E* respectively, and the network with *G*=(*V*,*E*). We denote the number of nodes as *n*=|*V*| and the number of edges as *m*=|*E*|. For each node *v*_*i*_, we denote its degree with *d*_*i*_.

Next, we define basic notations for probabilistic networks. We denote a probabilistic network as a graph $\mathcal {G}=(\mathcal {V},\mathcal {E},P)$, where $\mathcal {V}$ and $\mathcal {E}$ represent the node and edge sets respectively. *P* is a function defined on $\mathcal {E}$ ($P:\mathcal {E} \to (0,1]$), which returns the probability value of each edge. For each edge *e*_*i*_ in $\mathcal {G}$, we denote the probability that *e*_*i*_ is present and absent with *p*_*i*_ and *q*_*i*_ respectively (i.e., *p*_*i*_+*q*_*i*_=1). We denote the set of all possible deterministic network topologies that $\mathcal {G}$ can yield with $\mathcal {D}(\mathcal {G})= \{G = (\mathcal {V},\mathcal {E}') | \mathcal {E}' \subseteq \mathcal {E}\}$. We denote the probability that one can observe a specific deterministic network $G \in \mathcal {D}(\mathcal {G})$ with

1 $$ \mathcal{P}(G | \mathcal{G})=\prod\limits_{e_{i} \in \mathcal{E}'} p_{i} \prod\limits_{e_{j} \in \mathcal{E}-\mathcal{E}'} q_{j}.  $$

We explain the shortest path counting problem on a hypothetical probabilistic network $\mathcal {G}$ with five nodes and seven edges (see Fig. 1a). Figures 1b and c present two deterministic instances of this network among all 2^7^=128 possibilities. One way to compute the number of shortest paths is to generate all possible deterministic network topologies, find the number of shortest paths on all of them, and accumulate the results normalizing them with the probabilities of each deterministic network. Such an exhaustive approach however is impossible to scale beyond very small networks. The challenge, which lies at the heart of this paper, is that when the given network is probabilistic, it yields an exponential number of deterministic network topologies. Thus, even if one can deal with one of those instances by adopting heuristic solutions, this approach will fail to solve this problem for all possible deterministic topologies. In this paper, we develop a method to tackle this problem. We next describe our method.

### Counting shortest paths in probabilistic networks

The probabilistic nature of biological networks makes it infeasible to directly extend the shortest path counting methods for deterministic networks to probabilistic networks. The main reason behind this limitation is that the existence of the shortest path between a pair of nodes is uncertain and thus a longer path has the probability to be a shortest path. For example, consider a source node *a* and a sink node *d* in the probabilistic network $\mathcal {G}$ in Fig. 1a. In one possible deterministic topology *G*_1_ (see Fig. 1b), a shortest path with length 1 (i.e., {(*a*,*d*)}) between nodes *a* and *d* exists. However in another deterministic topology *G*_2_ (see Fig. 1c), two paths with length 3 (i.e., {(*a*,*c*),(*c*,*e*),(*e*,*d*)} and {(*a*,*c*),(*c*,*b*),(*b*,*d*)}) become the shortest paths as the edge directly linking nodes *a* and *d* is absent. Thus, in probabilistic networks, the number of shortest paths is uncertain and depends on the probability of edges constituting the paths between source and sink nodes.

Given a probabilistic network $\mathcal {G}=(\mathcal {V},\mathcal {E},P)$, we represent the number of shortest paths connecting nodes *s* and *t* with a random variable *B*_*s*∼*t*_. Let us denote the number of paths of length *k* between *s* and *t* in the *deterministic* network $G = (\mathcal {V},\mathcal {E})$, with $\pi _{s,t}^{k}$. Let us denote the frequency of the most abundant path length with $\pi _{s,t}^{*} = \max _{k} \left \{\pi _{s,t}^{k}\right \}$. The sample space for *B*_*s*∼*t*_ is the set of integers $\Omega = \left \{0, 1, \dots, \pi _{s,t}^{*}\right \}$. For example, given the probabilistic network in Fig. 1a, the sample space for *B*_*a*∼*d*_ is *Ω*={0,1,2} since there are at most two paths of lengths three and four (e.g., The two paths of length three are {(*a*,*c*),(*c*,*e*),(*e*,*d*)} and {(*a*,*c*),(*c*,*b*),(*b*,*d*)}). For each possible deterministic network topology $G \in \mathcal {D}(\mathcal {G})$, we define an indicator function *φ*(*G*,*k*). It takes the value 1 if there exists *k* shortest paths between source and sink nodes in network *G* and 0 otherwise. Thus the distribution of *B*_*s*∼*t*_ is defined by

$$Pr\left(B_{s \sim t}=k\right)=\underset{G \in \mathcal{D}(\mathcal{G})}{\Sigma}\varphi(G, k) \cdot \mathcal{P}(G| \mathcal{G}).$$

Computing the distribution of *B*_*s*∼*t*_ is a challenging task. This is because as the network topology is uncertain, any path between nodes *s* and *t* has a nonzero probability to be a shortest path. This depends on which possible paths are observed in a given deterministic network instance. Thus there is dependency between the existence probability of each path with those of remaining paths. There are two reasons behind this dependency. First, an edge can be shared by multiple paths. As a result, the absence/presence of this edge affects the absence/presence of all those paths containing it at the same time. Second, even when two different paths share no edges, the absence/presence of the shorter one affects the relevance of the other. This is because if the shorter one is present, then the other cannot be the shortest path. Next we provide an overview on how we calculate the distribution of *B*_*s*∼*t*_.

We model the dependency between a set of paths using a polynomial model. Our solution works in three steps. First, it locates all simple paths between nodes *s* and *t* in the deterministic network $\bar {G}=(\mathcal {V},\mathcal {E})$ which inherits all nodes and edges from $\mathcal {G}$ and assumes that all edges in $\mathcal {E}$ exist. We do this using a recursive depth-first search method. It then captures the dependency between paths connecting nodes *s* and *t* through the set of edges contributing to those paths. To do this, we build an undirected *bipartite graph*. After building this graph, we develop a new polynomial class, called the x-polynomial to express the contribution of the edges to the value of *B*_*s*∼*t*_ as imposed by this bipartite graph. We use this polynomial to compute the distribution of *B*_*s*∼*t*_.

#### Building the bipartite graph

We first discuss in detail how we capture the dependency between a set of paths with the help of a bipartite graph. Consider a probabilistic network $\mathcal {G}$. For each pair of nodes *s* and *t*, we denote the set of all possible paths between *s* and *t* with $\mathcal {H}_{s,t}$. For a given path $H_{i} \in \mathcal {H}_{s,t}$, let us denote the set of edges in *H*_*i*_ with $\mathcal {E}\left (H_{i}\right)$. Let us express the bipartite graph we construct with $\mathcal {G}_{s,t}=\left (\mathcal {V}_{1}, \mathcal {V}_{2}, M\right)$, where $\mathcal {V}_{1}$ and $\mathcal {V}_{2}$ represent the set of nodes and *M* represents the edges in $\mathcal {G}_{s,t}$ respectively. Each path $H_{i} \in \mathcal {H}_{s,t}$ corresponds to a node in $\mathcal {V}_{1}$. Each edge in $\bigsqcup _{H_{i} \in \mathcal {H}} \mathcal {E}(H_{i})$ corresponds to a node in $\mathcal {V}_{2}$. We insert an edge between nodes $u \in \mathcal {V}_{1}$ and $v \in \mathcal {V}_{2}$ if the path denoted by *u* contains the edge denoted by *v*. For instance, in Fig. 1a, consider the two nodes *a* and *d*. There are five simple paths between nodes *a* and *d*, $\mathcal {H}_{a,d}=\{H_{1}=\{(a,d)\},H_{2}=\{(a,c),(c,b),(b,d)\},H_{3}=\{(a,c),(c,e),(e,d \},H_{4}=\{(a,c),(c,b),(b,e),(e,d)\},H_{5}=\{(a,c),(c,e),(e,b),(b,d)\} \}$. Figure 1d shows the bipartite graph we construct for $\mathcal {H}_{a,d}$.

#### Computing the x-polynomial

We next discuss how we build the x-polynomial using the bipartite graph $\mathcal {G}_{s,t}=(\mathcal {V}_{1}, \mathcal {V}_{2}, M)$ for $\mathcal {H}_{s,t}$. This polynomial models all possible scenarios (i.e., subsets of feasible paths in $\mathcal {H}_{s,t}$) which can arise as a result of absence/presence of the edges denoted by $\mathcal {V}_{2}$. For each node $v_{i} \in \mathcal {V}_{1}$, we define a unique variable *x*_*i*_. For each node $v_{j} \in \mathcal {V}_{2}$, we represent its corresponding edge’s probability of presence and absence with *p*_*j*_ and *q*_*j*_ respectively (i.e., *p*_*j*_+*q*_*j*_=1). Using these notations, for each node $v_{j} \in \mathcal {V}_{2}$, we construct a polynomial, called the *edge polynomial*, and denote it with *Z*_*j*_. Formally

2 $$  Z_{j}= p_{j} \prod\limits_{\left(v_{i},v_{j}\right) \in M} x_{i} + q_{j}.  $$

This edge polynomial contains two terms. The first term is the product of the variables corresponding to all paths which contain that edge. The coefficient of this term is the probability of the presence of this edge. The second term only contains the probability of this edge being absent. To better understand edge polynomial, we explain it on the bipartite graph in Fig. 1d. Consider the node *e*_4_. There are four nodes connected to it, which are *H*_2_, *H*_3_, *H*_4_ and *H*_5_. Thus, the edge polynomial of *e*_4_ is (*p*_4_*x*_2_*x*_3_*x*_4_*x*_5_+*q*_4_). The first term of this polynomial represents the case that when edge *e*_4_ is present, it contributes to the existence of all four paths with a probability *p*_4_. The second term represents the case that if edge *e*_4_ is missing, none of four paths exists with a probability *q*_4_.

Now we are ready to define the x-polynomial which lies at the heart of our algorithm. As we explain later in this section, the x-polynomial provides a precise solution to the distribution of the random variable *B*_*s*∼*t*_.

##### **Definition 1**

Given a set of paths $\mathcal {H}_{s,t}$ between nodes *s* and *t*, and its bipartite graph $\mathcal {G}_{s,t}=(\mathcal {V}_{1}, \mathcal {V}_{2}, M)$, we denote the x-polynomial of $\mathcal {H}_{s,t}$ with $\mathcal {Z}_{s,t}$ and compute it as the product of all edge polynomials,

3 $$ \mathcal{Z}_{s,t}=\prod\limits_{v_{j} \in \mathcal{V}_{2}} Z_{j}.  $$

Notice that the x-polynomial of a given bipartite graph $\mathcal {G}_{s,t}=(\mathcal {V}_{1}, \mathcal {V}_{2}, M)$ contains $2^{|\mathcal {V}_{2}|}$ terms, with each term describing one possible deterministic network scenario for the edges in $\mathcal {V}_{2}$. Thus, the x-polynomial models all possible deterministic network topologies for the edges denoted by $\mathcal {V}_{2}$. Consider the bipartite graph in Fig. 1d. The x-polynomial is

$$\begin{array}{*{20}l} \mathcal{Z}_{a,d}&=\left(p_{1}x_{1}+q_{1}\right)\left(p_{2}x_{2}x_{4}+q_{2}\right)\\ &\left(p_{3}x_{2}x_{5}+q_{3}\right)\left(p_{4}x_{2}x_{3}x_{4}x_{5}+q_{4}\right)\\ &\left(p_{5}x_{3}x_{5}+q_{5}\right)\left(p_{6}x_{4}x_{5}+q_{6}\right)\left(p_{7}x_{3}x_{4}+q_{7}\right). \end{array} $$

Consider one of the terms resulting from the product of all edge polynomials in $\mathcal {Z}_{a,d}$, for example, $p_{1}p_{2}p_{3}p_{4}q_{5}q_{6}q_{7}x_{1}x_{2}^{3}x_{3}x_{4}^{2}x_{5}^{2}$. This term represents one possible deterministic topology where edges *e*_1_, *e*_2_, *e*_3_ and *e*_4_ are present with probability *p*_1_*p*_2_*p*_3_*p*_4_ while other edges are absent with probability *q*_5_*q*_6_*q*_7_.

In order to compute the distribution of *B*_*s*∼*t*_ from the x-polynomial, we introduce an operator called *collapse operator*. As we explain later, this operator will reduce the number of terms in the x-polynomial. It will also provide a representation of the number and the length of the paths.

For each node $v_{i} \in \mathcal {V}_{1}$, we denote its corresponding path’s length with *l*_*i*_. We also denote its degree with *deg*(*v*_*i*_) (i.e., *deg*(*v*_*i*_)=*l*_*i*_). This value indicates that during the construction of x-polynomial, at each term of the x-polynomial, variable *x*_*i*_ has an integer exponent ranging from 0 to *deg*(*v*_*i*_). Thus, we can write the *j*th term of the x-polynomial in the form $\alpha _{j}\prod \limits _{v_{i} \in \mathcal {V}_{1}}x_{i}^{c_{ij}}$, where *α*_*j*_ is the probability and *c*_*ij*_ is the exponent of the *i*th variable. For each variable *x*_*r*_, we define an indicator function *ψ*_*r*_(*c*). It takes the value 1 if *c*=*deg*(*v*_*r*_) and 0 otherwise. Using these notations, we define the collapse operator next.

*Collapse operator.* For each variable *x*_*r*_, we define a collapse operator *ϕ*_*r*_. This operator takes a term of the x-polynomial as the input. Let us assume the input is the *j*th term. We define the collapse operator as

4 $$ {\begin{aligned} \phi_{r}\left(\alpha_{j} \prod\limits_{v_{i} \in \mathcal{V}_{1}} x_{i}^{c_{ij}}\right) = \alpha_{j} \left[t_{l_{r}} \psi_{r}\left(c_{rj}\right)+\left(1-\psi_{r}\left(c_{rj}\right)\right)\right] \prod\limits_{v_{i} \in \mathcal{V}_{1} \setminus v_{r}} x_{i}^{c_{ij}}. \end{aligned}}  $$

Notice that the collapse operator *ϕ*_*r*_ only alters the variable *x*_*r*_ in each term of the x-polynomial. It has two possible outcomes. (1) If *ψ*_*r*_()=1, it means that all edges in the path *H*_*r*_ are present so that a path with length *l*_*r*_ exists. In that case, we replace *x*_*r*_ with the variable $t_{l_{r}}$. (2) If *ψ*_*r*_()=0, it means that at least one edge in the path *H*_*r*_ is missing. Thus, the entire *H*_*r*_ is missing. In that case, we remove the term *x*_*r*_.

To understand the collapse operator better, consider one term of the x-polynomial $\mathcal {Z}_{a,d}$ (see above), namely $p_{1}p_{2}p_{3}p_{4}q_{5}q_{6}q_{7}x_{1}x_{2}^{3}x_{3}x_{4}^{2}x_{5}^{2}$. Applying *ϕ*_1_() to this term, replaces the variable *x*_1_ with *t*_1_ as *deg*(*H*_1_)=1 and the exponent of *x*_1_ in this term is 1. Similarly, applying *ϕ*_2_() to this term, transforms it to $p_{1}p_{2}p_{3}p_{4}q_{5}q_{6}q_{7}t_{1}t_{3}x_{3}x_{4}^{2}x_{5}^{2}$ as *deg*(*H*_2_)=3 and the exponent of *x*_2_ is 3. Finally, applying *ϕ*_3_(), *ϕ*_4_() and *ϕ*_5_() to this term, eliminates the variables *x*_3_, *x*_4_ and *x*_5_ as their exponents are smaller than the degrees of their corresponding nodes in the bipartite graph. Thus, the term gets simplified to only two variables (i.e., *p*_1_*p*_2_*p*_3_*p*_4_*q*_5_*q*_6_*q*_7_*t*_1_*t*_3_).

Next, we discuss when we apply the collapse operator. Without violating the generality of the discussion, let us assume that we multiply the edge polynomials in the order $Z_{1}, Z_{2}, \dots, Z_{|\mathcal {V}_{2}|}$. We say that *Z*_*j*_ is the *final edge polynomial of the variable* *x*_*r*_ in this ordering if the following two conditions hold: 1) *Z*_*j*_ contains variable *x*_*r*_, and 2) ∀*i*>*j*, *Z*_*i*_ does not contain *x*_*r*_. We apply the collapse operator *ϕ*_*r*_() to the resulting polynomial terms as soon as we finish multiplying the final edge polynomial of *x*_*r*_. This is because multiplying the remaining edge polynomials does not alter the exponent of *x*_*r*_. Thus, each term at this point either denotes the presence or absence of the path *H*_*r*_.

After multiplying all edge polynomials and collapsing, the x-polynomial $\mathcal {Z}_{s,t}$ takes the following form:

5 $$  \mathcal{Z}_{s,t}=\sum\limits_{j} \alpha_{j} \prod\limits_{i} t_{i}^{c_{ij}}.  $$

Notice that the *j*th term of the x-polynomial lists the number of paths (i.e., *c*_*ij*_) for each path length (i.e., *i*) and the existence probability of these paths (i.e., *α*_*j*_). We observe that one term may contain multiple paths with different length. For example, the term *p*_1_*p*_2_*p*_3_*p*_4_*q*_5_*q*_6_*q*_7_*t*_1_*t*_3_ describes a deterministic topology which contains two paths connecting a pair of nodes, one path with length 1 and another with length 3. Notice that, given the existence of a path of length 1, the existence of path of length 3 becomes irrelevant as the former path is shorter. In order to count the number of shortest paths, for each term of the x-polynomial, we remove all paths whose lengths are not the smallest. To do that, we introduce an operator called *select operator*. Let *ζ*_*j*_(*t*_*r*_) represent an indicator function for each variable *t*_*r*_ in the *j*th term. *ζ*_*j*_(*t*_*r*_)=1 if path with length *r* is shortest in the specific deterministic network topology described by the *j*th term, and 0 otherwise. We denote a shortest path by variable *y*. Using these notations, we define our select operator next.

*Select operator.* For the *j*th term of the x-polynomial (i.e., $\alpha _{j} \prod _{i} t_{i}^{c_{ij}}$), we define the select operator *δ*_*j*_() as:

6 $$\begin{array}{*{20}l} \begin{aligned}  \delta_{j}\left(\alpha_{j} \prod\limits_{i} t_{i}^{c_{ij}}\right) =& \alpha_{j}\prod\limits_{i} \left(\zeta_{j}\left(t_{i}\right)y + \left(1-\zeta_{j}\left(t_{i}\right)\right)\right)^{c_{ij}} \\ =& \alpha_{j} y^{c_{j}}. \end{aligned} \end{array} $$

Assume that in the *j*th term, the path with length *r* is the shortest path. Thus, in the above equation, *c*_*j*_ equals to the exponent of variable *t*_*r*_ (that is *c*_*j*_=*c*_*rj*_). Notice that select operator only replace *t*_*r*_ with variable *y* while removing other *t*_*i*_ (i.e, *i*>*r*). For example, given that the term *p*_1_*p*_2_*p*_3_*p*_4_*q*_5_*q*_6_*q*_7_*t*_1_*t*_3_ contains *t*_1_, by applying select operator, we remove *t*_3_ and replace *t*_1_ with *y* further simplifying this term to *p*_1_*p*_2_*p*_3_*p*_4_*q*_5_*q*_6_*q*_7_*y*. Notice that, the select operator can also track the length of the shortest path for each term if we use *y*_*r*_ to represent the shortest path in the *j*th term instead of *y*. As we only count the number of shortest paths in this paper, for simplicity, we only use *y* to represent the shortest path.

After applying select operator, the final x-polynomial takes the following form:

7 $$  \mathcal{Z}_{s,t}=\sum\limits_{k=0}^{\pi_{s,t}^{*}} p_{k} y^{k}.  $$

##### **Theorem 1**

Consider a probabilistic graph $\mathcal {G}$, two nodes *s* and *t* in $\mathcal {G}$, the set of all paths $\mathcal {H}_{s,t}$ between *s* and *t*, and its collapsed polynomial $\mathcal {Z}_{s,t}$. The coefficients of the polynomial $\mathcal {Z}_{s,t}$ are the true distribution of the random variable *B*_*s*∼*t*_.

**Proof.** We focus on the *j*th term $\phantom {\dot {i}\!}\alpha _{j} y^{c_{j}}$ after applying select operator. By its definition, it generates a term containing *c*_*j*_ shortest paths between nodes *s* and *t*. Thus, after adding the coefficients of all the terms *y*^*k*^, *p*_*k*_ equals the probability that exactly *k* shortest paths exist between nodes *s* and *t*. Recall that *B*_*s*∼*t*_ takes an integer value in the [0, $\pi _{s,t}^{*}$] interval. The probability distribution values of *B*_*s*∼*t*_ which are corresponding to $[0,1,\dots,\pi _{s,t}^{*}]$ are $[p_{0}, p_{1}, \dots, p_{\pi _{s,t}^{*}}]$. □

Once we have the probability distribution of *B*_*s*∼*t*_, we can characterize the number of shortest paths precisely.

##### **Corollary 1**

Consider a probabilistic graph $\mathcal {G}$, two nodes *s* and *t* in $\mathcal {G}$, the set of all paths $\mathcal {H}_{s,t}$ between *s* and *t*, and its collapsed polynomial $\mathcal {Z}_{s,t}$. The expected number of shortest paths between nodes *s* and *t* is

8 $$ \text{Exp}\left(B_{s \sim t}\right)=\sum\limits_{k = 0}^{\pi_{s,t}^{*}} k\times p_{k}.  $$

We use the expected number of shortest paths to quantify the number of shortest paths between a pair of nodes in our experiment.

**Implementation Details.** Recall that the select operator requires identifying the shortest paths while removing other longer paths. In our implementation, however we develop a two-step solution to avoid generating such variables *t*_*j*_ when there exists another variable *t*_*i*_ with *i*<*j*. The key idea of our two-step solution is that we apply the select operator whenever possible. For all possible path length of paths between source and sink nodes, we rank them in ascending order. Without violating the generality of the discussion, let us assume that we multiply the edge polynomials in the order $Z_{1}, Z_{2}, \dots, Z_{|\mathcal {V}_{2}|}$. Based on this ordering, for the current smallest path length (say *k*) to be processed, we say *Z*_*j*_ is the *final edge polynomial of path length k* if the following two conditions hold: 1) *Z*_*j*_ contains at least one variable *x*_*r*_ with *deg*(*v*_*r*_)=*k*; 2) ∀*i*>*j*, all variables in *Z*_*i*_ have *deg*()>*k*.

We apply the select operator to all polynomial terms containing *t*_*k*_ as soon as we complete the multiplication and collapsation of the final edge polynomial of path length *k*. The rationale behind this is that given that the current shortest path length is *k*, after multiplying the final edge polynomial of path length *k*, if one term already has variable *t*_*k*_ (i.e., already has at least one path with length *k*), the longer paths generated by multiplying following edge polynomials can not be the shortest paths any more. We discuss this solution in detail next.

First, we order the nodes in $\mathcal {V}_{1}$ in ascending order of their degrees, and group nodes with the same degree. Let us denote the subset of nodes of $\mathcal {V}_{1}$ with degree *r* with *S*_*r*_. For example, in Fig. 1d, five paths can be divided into three groups. *S*_1_={*H*_1_} as the degree of *H*_1_ is 1. Similarly, *S*_3_={*H*_2_,*H*_3_} and *S*_4_={*H*_4_,*H*_5_} (i.e., the degree of them are 3 and 4 respectively).

In the second step, we iteratively consider the groups *S*_*r*_ in ascending order of *r*. At each iteration, we multiply the edge polynomials associated with the nodes of the group *S*_*r*_ considered at that iteration as follows. For each group *S*_*r*_, its associated edge polynomials are $\bigsqcup _{v_{i} \in S_{r}, (v_{i},v_{j}) \in M} \{Z_{j}\}$. We iterate over the edge polynomials in this set. For each edge polynomial, if it has not been multiplied before, we multiply it with the existing product and collapse it if it is the final edge polynomial of some *x* variables; otherwise, we skip it. Thus, the last multiplied edge polynomial in this set is the final edge polynomial of path length *r*. Once we complete the iterations for *S*_*r*_, we apply the select operators to the resulting polynomial terms. In doing that, we consider the terms in two different categories. Those, that contain the variable *t*_*r*_ constitute the first category. The remaining terms make up the second category. We only apply the select operator on the terms in the first category, in which we remove all *x*_*i*_ variables and replace *t*_*r*_ with variable *y*. We also do not multiply terms in the first category with any other edge polynomial in the subsequent iterations. We do this to prevent the formation of paths longer than *r* as we are ensured to have at least one path of length *r* for those terms. The following example demonstrates how our strategy works.

### Example of the counting shortest paths strategy

Consider the bipartite graph in Fig. 1d. This graph has three groups of nodes in $\mathcal {V}_{1}$, namely *S*_1_, *S*_3_, and *S*_4_. We first deal with the edge polynomials of *S*_1_. Since *Z*_1_=*p*_1_*x*_1_+*q*_1_ is the only edge polynomial, we apply the collapse operator *ϕ*_1_(). After collapsing, we obtain two terms *p*_1_*t*_1_ and *q*_1_. Notice that the first term *p*_1_*t*_1_ indicates that there is a shortest path of length one with probability *p*_1_. Given *p*_1_*t*_1_ contains *t*_1_ belonging to the first category, we apply select operator on it and do not consider this term for further polynomial multiplication. We only use the other term *q*_1_ for further multiplication. Next, we consider the group *S*_3_={*H*_2_,*H*_3_}. Its associated edge polynomials are *Z*_2_, *Z*_3_, *Z*_4_, *Z*_5_ and *Z*_7_. We multiply these polynomials with the existing one (i.e., simply *q*_1_). We then apply the collapse operators *ϕ*_2_() and *ϕ*_3_(). This process will introduce new terms containing variables *t*_3_. We then apply select operator on all such terms as they fall into the first category showing the probability that the shortest path length is three. Finally, we focus on group *S*_4_={*H*_4_,*H*_5_}. Although its associated edge polynomial set contains all edge polynomials (except *Z*_1_), we only multiply *Z*_6_ as remaining ones have already been multiplied in the previous iterations. We then collapse the resulting polynomial using operators *ϕ*_4_() and *ϕ*_5_() to obtain the terms containing *t*_4_. This way, we aggregate all possible scenarios of shortest path lengths.

We observe that the terms applying select operator early (i.e., the terms constituting the first category) will not attend the following edge polynomial multiplication, which may have the potential to generate incorrect polynomial terms. Following lemma proves that the terms applying select operator early are exactly same with those generated after multiplying all edge polynomials, collapsing and then applying select operator. We start by defining our notation. We denote the function to apply all collapse operators to polynomial terms with *Φ*(). Now we are ready to prove the correctness of our two-step solution.

#### **Lemma 1**

Consider a probabilistic graph $\mathcal {G}$, two nodes *s* and *t* in $\mathcal {G}$, and the set of all paths $\mathcal {H}_{s,t}$ between *s* and *t*. Given the current smallest unprocessed path length k, after multiplying the final edge polynomial *Z*_*r*_ of path length *k*, consider the collapsed polynomial $\mathcal {Z}_{s,t} = \left (\underset {j}{\Sigma } \alpha _{j} t_{k}^{c_{kj}} \prod \limits _{i > k} t_{i}^{c_{ij}} \prod \limits _{i} x_{i}^{c_{ij}}\right) + \underset {j}{\Sigma } \alpha _{j} \prod \limits _{i > k} t_{i}^{c_{ij}} \prod \limits _{i} x_{i}^{c_{ij}}$. Assume that the remaining unprocessed edge polynomial set is *S*. Applying select operator to terms containing *t*_*k*_ (i.e., the first term constitutes the first category) outputs the exact same terms as those generated after multiplying all remaining edge polynomials, collapsing and then applying select operator. Mathematically

$$\begin{array}{*{20}l} &\underset{j}{\Sigma} \delta_{j}\left(\alpha_{j} t_{k}^{c_{kj}} \prod\limits_{i > k} t_{i}^{c_{ij}} \prod\limits_{i} x_{i}^{c_{ij}}\right) \\ =&\underset{j}{\Sigma} \delta_{j}\left(\Phi\left(\alpha_{j} t_{k}^{c_{kj}} \prod\limits_{i > k} t_{i}^{c_{ij}} \prod\limits_{i} x_{i}^{c_{ij}}\prod\limits_{Z_{i} \in S} Z_{i}\right)\right). \end{array} $$

**Proof.** We focus on the *j*th term containing *t*_*r*_, $\alpha _{j} t_{k}^{c_{kj}} \prod \limits _{i > k} t_{i}^{c_{ij}} \prod \limits _{i} x_{i}^{c_{ij}}$. After applying select operator, we obtain

9 $$  \delta_{j}\left(\alpha_{j} t_{k}^{c_{kj}} \prod\limits_{i > k} t_{i}^{c_{ij}} \prod\limits_{i} x_{i}^{c_{ij}}\right) = \alpha_{j} y^{c_{kj}}.  $$

Now, we first multiply the *j*th term with the remaining edge polynomials, collapse and then apply select operator, we obtain

10 $$\begin{array}{*{20}l}  \begin{aligned} &\delta_{j}\left(\Phi\left(\alpha_{j} t_{k}^{c_{kj}} \prod\limits_{i > k} t_{i}^{c_{ij}} \prod\limits_{i} x_{i}^{c_{ij}} \prod\limits_{Z_{i} \in S} Z_{i}\right)\right) \\ =& \delta_{j}\left(\alpha_{j} t_{k}^{c_{kj}} \prod\limits_{i > k} t_{i}^{c_{ij}} \Phi\left(\prod\limits_{i} x_{i}^{c_{ij}} \prod\limits_{Z_{i} \in S} Z_{i}\right)\right) \end{aligned} \end{array} $$

We expand the product of edge polynomials, and rewrite $\Phi \left (\prod \limits _{i} x_{i}^{c_{ij}} \prod \limits _{Z_{i} \in S} Z_{i}\right)$ as

$$\begin{array}{*{20}l} &\Phi\left(\prod\limits_{i} x_{i}^{c_{ij}} \prod\limits_{Z_{i} \in S} Z_{i}\right) \\ =&\Phi\left(\prod\limits_{i} x_{i}^{c_{ij}} \prod\limits_{Z_{i} \in S} \left(p_{i}\prod\limits_{(v_{k},v_{i}) \in M}x_{k} + q_{i}\right)\right) \\ =& \Phi\left(\Sigma_{r} \beta_{r} \prod\limits_{i} x_{i}^{c_{ir}}\right) \\ =& \Sigma_{r} \beta_{r} \prod\limits_{i > k}t_{i}^{c_{ir}} \end{array} $$

Here, *β*_*r*_ is the coefficient of the *r*th polynomial term. Notice that after multiplying the final edge polynomial of length *k*, multiplying the remaining edge polynomials and collapsing leads to only variables *t*_*i*_ (*i*>*k*). We inject the above equation to Eq. 10, we obtain

$$\begin{array}{*{20}l} \begin{aligned} &\delta_{j}\left(\alpha_{j} t_{k}^{c_{kj}} \prod\limits_{i > k} t_{i}^{c_{ij}} \Phi\left(\prod\limits_{i} x_{i}^{c_{ij}} \prod\limits_{Z_{i} \in S} Z_{i}\right)\right) \\ =& \delta_{j}\left(\alpha_{j} t_{k}^{c_{kj}} \prod\limits_{i > k} t_{i}^{c_{ij}} \Sigma_{r} \beta_{r} \prod\limits_{i > k}t_{i}^{c_{ir}}\right) \\ =&\alpha_{j} y^{c_{kj}} \Sigma_{r} \beta_{r} \end{aligned} \end{array} $$

Notice that *Σ*_*r*_*β*_*r*_ equals to the sum of coefficients of product of edge polynomials, that is

$$\Sigma_{r} \beta_{r} = \prod\limits_{Z_{i} \in S} \left(p_{i} + q_{i}\right) = 1.$$

Thus

$$\delta_{j}\left(\Phi\left(\alpha_{j} t_{k}^{c_{kj}} \prod\limits_{i > k} t_{i}^{c_{ij}} \prod\limits_{i} x_{i}^{c_{ij}} \prod\limits_{Z_{i} \in S} Z_{i}\right)\right) = \alpha_{j} y^{c_{kj}}.$$

□

Theorem 1 proves that the final x-polynomial generated by multiplying and collapsing all edge polynomials, and applying the select operator in the end, does generate the true distribution of the random variable *B*_*s*∼*t*_. Different from this method, our two step solution however applies the select operator to some terms whenever possible and prevents their multiplication with remaining edge polynomials. Notice that Lemma 1 proves that applying select operator early to those polynomial terms making up the first category does output the same results with those generated by applying select operator to final polynomial terms. Thus, our two step solution outputs the true distribution of the random variable *B*_*s*∼*t*_. We test the correctness of this proof on the probabilistic network in Fig. 1. Recall that the set of paths between nodes *a* and *d* in this figure are $\mathcal {H}_{a,d}=\{H_{1}=\{(a,d)\},H_{2}=\{(a,c),(c,b),(b,d)\},H_{3}=\{(a,c),(c,e),(e,d \},H_{4}=\{(a,c),(c,b),(b,e),(e,d)\},H_{5}=\{(a,c),(c,e),(e,b),(b,d)\} \}$. Using our two step strategy, we obtain $\mathcal {Z}_{a,d} = 0.8270352 + 0.1517968y+0.021168y^{2}. $ Notice that the coefficient of *y*^*k*^ is the probability that exactly *k* shortest paths exist. In this figure, in order to have exactly two shortest paths, the paths *H*_2_ and *H*_3_ must exist and the path *H*_1_ must be absent. This is because *H*_2_ and *H*_3_ have the same length (i.e., three), and *H*_1_ is shorter than those two paths. This condition is satisfied only when edges *Set*_1_={(*a*,*c*),(*c*,*b*),(*b*,*d*),(*c*,*e*),(*e*,*d*)} exist and the edge *Set*_2_={(*a*,*d*)} does not exist. The absence/presence of the edge (*b*,*e*) has no influence on the outcome. Multiplying the existance probabilities of the edges in *Set*_1_ and the absence probabilities of the edge in *Set*_2_ yields the coefficient of *y*^2^ as calculated by our algorithm.

## Results

In this section, we evaluate performance of our shortest path counting method and its application to identify communities in networks on both synthetic and real datasets. We compare our method’s accuracy and computational cost to three existing approaches, namely *binary*, *threshold*, and *sampling* methods. Next we describe the datasets we use in our experiments and the methods we compare against.

*Datasets* In this section we describe the synthetic and real datasets we used in our experiments.

**Synthetic datasets.** To observe the performance of our method under controlled dataset characteristics, we perform extensive experiments on synthetically generated directed networks. In the following, to simplify our notation, we use the *size* and *average degree* of the network to represent the number of nodes and the number of edges per node in a network respectively. We run experiments on synthetic directed networks under three varying parameters; network size, average degree, and the probability model. To do this, we generate LFR benchmark networks [38]. This benchmark uses several parameters in constructing networks. We set the exponents of the degree distribution and the community size distribution to 2 and 1 respectively. We fix the mixing parameter *μ* to 0.2, which means that each node shares a fraction 1−*μ* of edges with the other nodes of its community and a fraction *μ* with the other nodes of other communities. We vary all the other parameters, such as number of nodes and average degree.

After generating LFR benchmark networks, we assign probability values to the edges of these networks using three probability models. These models include identical, uniform and normal distribution models. For the identical model, we assign each edge with the same probability value. Under the uniform and normal distribution models, we assign each edge with a random number in the (0,1] interval generated from the uniform and normal distribution respectively. We vary the standard deviation to observe the impact of variation in probability values.

**Real datasets.** We analyze the cell cycle network of different cancer types from KEGG database using our shortest path counting and community detection methods. The network contains 60 nodes and 83 edges. We use five cancer datasets, including the prostate cancer dataset from the The Cancer Genome Atlas (TCGA), and breast cancer (GSE50948), colon cancer (GSE17536), lung cancer (GSE19804) and leukemia cancer (GSE71014) datasets from NCBI. For each cancer dataset, we build its corresponding cell cycle network where the probability of each interaction within the network is equal to the correlation value between corresponding genes. Thus, we have five versions of the cell cycle network.

**Competing methods.** Recall from the “Background” section that current approaches to the probabilistic networks often transform probabilistic networks to deterministic networks first, and then apply methods developed specifically for deterministic networks. These approaches include ignoring probability values [34, 35], considering edges with probability values above a given threshold [37], and sampling the probabilistic network by doing a Bernoulli trial with probability *p*_*i*_ for each edge *e*_*i*_ [36]. We call these three approaches as binary, threshold, and sampling methods, respectively. For these three methods, after transforming probabilistic networks to deterministic networks, we calculate the number of shortest paths. To detect communities in biological networks, we apply Newman and Grivan method [39] on the deterministic networks. Please note that finding the shortest path between two nodes in a probabilistic network comprises finding the path with minimum length, which utilizes a nonlinear function (see “Shortest path counting utilizes a nonlinear function” section for details). Although, a sampling approach can provide provable confidence intervals for estimating linear functions such as sum and average, it fails to do that for nonlinear functions such as finding the minimum. Due to the nonlinear nature of our problem a sampling approach is expected to produce inaccurate results even when a large number of samples are used.

### Accuracy and computational cost of shortest path counting methods

In this section, we evaluate accuracy and speed of our shortest path counting method on synthetic networks under three parameters; network size, average degree and probability model. To observe the effect of each parameter, in each experiment we vary only one parameter while fixing the others. To ensure the reliability of our results, for each parameter, we conduct experiments on 10 different networks and report the median. Specifically, for sampling method, to get a reliable result, for each network, we sample the network 1000 times and count the average number of shortest paths.

Our shortest path counting method is exact (see “Methods” section for proof), and thus we use it here as the reference. We calculate the aforementioned three existing methods’ relative error in comparison to our method as $\frac {|f-f^{*}|}{f^{*}}$, where *f*^∗^ and *f* represent the number of shortest paths found between two nodes by using our method (i.e., true value) and one of the three existing methods, respectively. Next, we test the effect of network size on accuracy and speed.

**Fig. 2:**
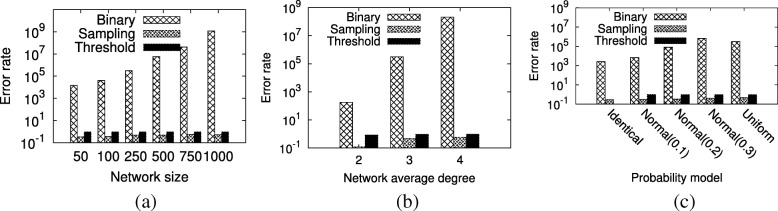
The accuracy of shortest path counting methods on synthetic networks with different network sizes (**a**), average degrees (**b**) and probability models (**c**)

*Effect of network size.* Here, we explore the impact of network size on accuracy of the three existing shortest path counting methods. We set the synthetic networks’ average degree to three and the probability model to uniform model. We experiment for network sizes 50, 100, 250, 500, 750 and 1000. Figure 2a reports the result. Our results demonstrate that binary method has massive relative error rate for all the networks. The threshold and sampling methods achieve better results than the binary method; yet their relative error rates are still substantial (around 95% and 45%, respectively). It is also worth noting that we give a positive bias towards the threshold method since we fix the threshold to 0.6, which minimizes its error rate in our experiments (see “Effect of the threshold value” section). We observe that the error rate in counting number of shortest paths for the binary method and sampling methods grow with network size, but error for the threshold method is slightly invariant. These results suggest that existing methods, which model probabilistic networks as deterministic ones, are grossly inaccurate for counting the number shortest paths. Our novel method on the other hand is provably precise for the same problem.

*Effect of average degree.* Next, we explore the impact of average node degree on shortest path counting. We use networks of average degree 2, 3 and 4. We fix the network size to 250 nodes and apply the uniform model to assign interaction probabilities. Figure 2b shows the results. We observe that the error rate for binary method is larger than the threshold and sampling methods. In comparison to our method, threshold and sampling methods incur up to 96% and 58% relative error values, respectively. We observe that the error rate increases for all methods as average degree increases. We conjecture this is probably due to the fact that as the number of edges increases, the number of shortest paths increases between pairs of nodes as well, leading to increase in error.

*Effect of probability model.* Finally, we focus on the impact of probability model on shortest path counting. For each network topology model, we generate 10 networks with size 250 and average degree 3. For each network, we assign probability values to interactions using three probability models: identical, uniform and normal distribution. Identical model sets the probability of all edges to 0.5. We use three different normal distribution models. All three models have the same mean (0.5), but varying standard deviations (0.1, 0.2 and 0.3). This way, we test a wide spectrum of possible variations in interaction probabilities ranging from no variation (identical model) to very large variation (normal distribution with 0.3 standard deviation). Notice that identical probability model is meaningless to apply when threshold method is used for shortest path counting. This is because either all edges are removed or all are retained depending on the threshold since all edges have identical probabilities. Therefore, we do not report the threshold method for the identical probability model. Figure 2c presents the results.

Our results demonstrate that binary and sampling methods produce the highest and lowest errors overall across different probability models, respectively. The threshold and sampling methods’ errors range between 95%-100% and 28%-48%, respectively. In the extreme case, when all probabilities are identical, we observe that binary method yields less error in comparison to when the edge probability values are heterogeneous. We would like to note that threshold and sampling methods are less sensitive to the distribution of the edge probabilities in comparison to the binary method. Thus they can adapt to variations in interaction probabilities better.

**Fig. 3:**
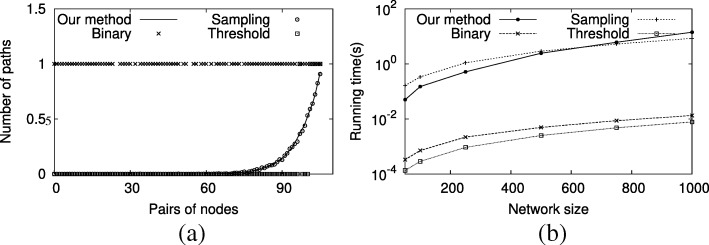
The distribution of number of paths (**a**) and computational cost (**b**) of shortest path counting methods on synthetic networks

Our analysis on synthetic networks demonstrate that existing shortest path counting methods for probabilistic networks are inaccurate. To discern the source of their failure, next, we plotted the distribution of number of paths across all pairs of nodes for our method and the three existing methods for a uniformly distributed probabilistic network of size 250 and average degree 4. Figure 3a shows the results. Please note that for illustrative purposes we ranked the gene pairs in regards to their number of paths in this plot. Our experiments demonstrate that existing shortest path counting methods either under or over estimates the number of shortest paths. We observe that usually the binary approach overestimates, and the threshold method underestimates the number of shortest paths for a given node pair. Although, random sampling approach provides somewhat similar results to our method, for some gene pairs it over or under estimates the number of shortest paths also. As we have mentioned before, sampling methods might provide quite accurate results for linear functions, however they do not work well for nonlinear functions such as shortest path counting (see “Shortest path counting utilizes a nonlinear function” section). Due to the nonlinear nature of our problem, sampling method leads to unreliable results in our experiments even if a large number of samples are used. These observations suggest that the right treatment of probabilistic interactions is essential for precisely counting the number of shortest paths in biological networks.

Finally, we evaluate the running time of our method. We experiment for network sizes varying from 50 to 1000. We set the average degree of these networks to three, and probability model to uniform model. Figure 3b presents the results. Our results suggest that binary and threshold methods’ computational times are comparable, and they take the least amount of time. Our method’s running time is similar to that of the sampling method. Both of these methods are slower than binary and threshold methods. In networks of varying sizes (50-1000) our method runs in 0.05-14.2 s. This suggests that although our method is slightly slower than binary and threshold methods, it is still very fast, and scalable to large scale biological networks. Next, we test our method on a real cancer dataset.

**Fig. 4:**
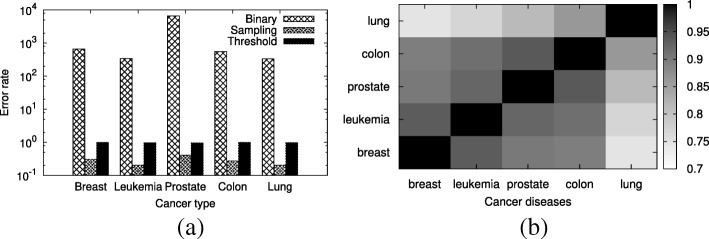
The accuracy of shortest path counting methods on real cancer networks (**a**), and the rand index of the community structures of different cancer types (**b**)

*Evaluation of shortest path counting algorithm on cancer networks.* In this experiment, first, we analyze the accuracy of the existing shortest path counting algorithms using cell cycle pathway in a few cancer types. Recall that the interaction probabilities can change across different cancer types. Our results on cancer networks coincide with those on the synthetic dataset (see Fig. 4a). Binary method is the least accurate method among the three existing approaches. Although threshold and random sampling approaches are more accurate than the binary method, their accuracies are much lower than that of our method. Threshold and random sampling approaches result in up to 100% and 40% relative errors in comparison to our method, respectively.

Next, using our method, we count the number of shortest paths for each cell cycle gene pair in five cancer datasets (breast, colon, leukemia, lung and prostate). We rank these genes according to the number of shortest paths between them and others. Our algorithm successfully ranks the CDK1 gene as the top gene for all cancer types analyzed here. CDK1 gene is known to be a key regulator of the cell cycle pathway, and it is a potential therapeutic target for inhibitors in cancer treatment. CDK1 gene’s major role in development and cancer is supported by many experimental studies [40–43]. Genetic substitution of CDK1 gene has been shown to cause embryonic lethality [44], and its inhibition has been suggested as a potential therapy for MYC-dependent breast cancer [41].

**Fig. 5:**
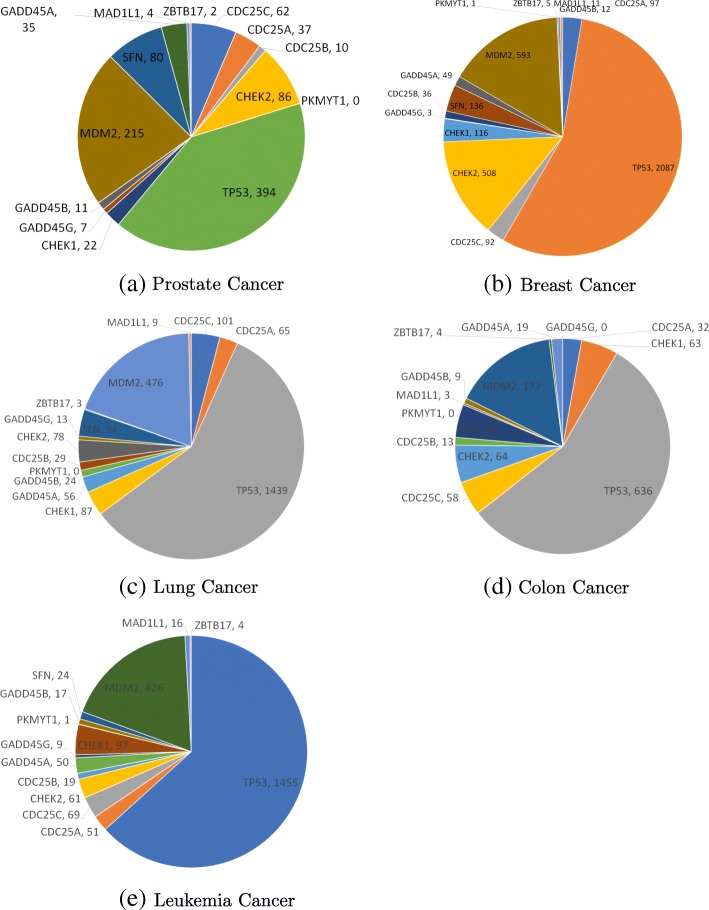
The publication count of genes appearing in the shortest paths of real canner networks

We also rank the genes using their expected node betweenness. Table 1 presents the result. As five cancer networks have the same network topology, we identify the same set of genes that appear in the shortest paths (i.e., node betweenness is greater than 0) but with different ranking. For these identified genes, we do the literature analysis as follows. Given a specific cancer type, for each gene on the shortest path, we count the number of publications in PubMed containing this gene and this specific cancer type. Figure 5 shows the results. We observe that all genes have a large number of publications related to specific cancer. Furthermore, we also observe that the percentage of publications of top 3 genes exceeds 50% in almost all cancer types, which suggests that the genes with high node betweenness have great potential to exhibit biological significance.

**Table 1:** The node betweenness of genes appearing in the shortest paths

	Prostate		Breast		Lung		Colon		Leukemia	
Ranking	A	B	A	B	A	B	A	B	A	B
1	CDC25C	1.83	CDC25A	0.37	CDC25C	1.87	CDC25A	2.20	TP53	3.00
2	CDC25A	1.51	TP53	0.35	CDC25A	1.66	CHEK1	0.59	CDC25A	2.12
3	CDC25B	0.88	CDC25C	0.33	TP53	1.24	TP53	0.56	CDC25C	1.53
4	CHEK2	0.61	CHEK2	0.24	CHEK1	0.62	CDC25C	0.47	CHEK2	1.15
5	PKMYT1	0.38	CHEK1	0.24	GADD45A	0.28	CHEK2	0.47	CDC25B	0.77
6	TP53	0.18	GADD45G	0.14	GADD45B	0.27	CDC25B	0.42	GADD45A	0.60
7	CHEK1	0.13	CDC25B	0.10	PKMYT1	0.23	SFN	0.24	GADD45G	0.52
8	GADD45G	0.06	SFN	0.05	CDC25B	0.20	PKMYT1	0.12	CHEK1	0.37
9	GADD45B	0.06	GADD45A	0.05	CHEK2	0.18	MAD1L1	0.11	PKMYT1	0.33
10	MDM2	0.04	MDM2	0.04	GADD45G	0.16	GADD45B	0.08	GADD45B	0.27
11	SFN	0.02	PKMYT1	0.03	SFN	0.15	MDM2	0.03	SFN	0.04
12	GADD45A	0.01	ZBTB17	0.02	ZBTB17	0.10	ZBTB17	0.02	MDM2	0.03
13	MAD1L1	0.00	MAD1L1	0.02	MDM2	0.02	GADD45A	0.02	MAD1L1	0.01
14	ZBTB17	0.00	GADD45B	0.00	MAD1L1	0.01	GADD45G	0.02	ZBTB17	0.01

A = gene name. B = node betweenness

In summary, our experiments on synthetic and real datasets demonstrate that existing methods either under or over estimate the number of shortest paths with a large margin. The binary method performs the worst among the existing three methods under varying network sizes, average node degrees and probability distributions. In comparison to the existing methods, our novel method solves the shortest path counting problem accurately in a feasible time. The low accuracy level of existing methods suggests that our novel method is truly needed in the field since the existing methods might potentially lead to wrong biological implications. Our analysis on real datasets support our findings on synthetic data, and suggest that our approach could be used to discover key genes in biological systems.

### Effects of network model parameters on community detection

In this section, we use our shortest path counting method to identify communities in biological networks. We use the same experimental set up from the previous section. We test the robustness of our community detection approach under varying network sizes, average node degrees and probability models. We run experiments on both synthetic and real cancer networks, and compare the expected modularity value of our method to three existing methods. We run experiments on synthetic networks and compare the expected modularity value of our method to three existing methods. To observe the effect of each parameter, in each experiment we vary only one parameter and fix the other two. To ensure the reliability of our results, for each parameter, we conduct experiments on 10 different networks and report the median. Specifically, for sampling method, we calculate the modularity by sampling the network 1000 times and report the average modularity.

**Fig. 6:**
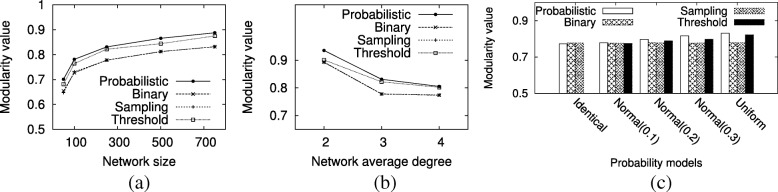
The expected modularity value of our method and other methods on synthetic networks with different network sizes (**a**), average degrees (**b**) and probability models (**c**)

*Effect of network size on community detection.* First, we explore the impact of network size. We set the average degree to three and the probability model to uniform model. We experiment for network sizes 50, 100, 250, 500 and 750. Figure 6a reports the result. Our results demonstrate that our method has the highest modularity value for all networks with over 50 nodes. The threshold method achieves the second best modularity value. That said, it is worth noting that we give a positive bias towards the threshold method since we fix the threshold to the value which maximized its modularity in our experiments (see “Effect of the threshold value” section). Sampling and binary methods obtain similar results. We observe that the expected modularity value of all methods grows with the network size. This is maybe because the effect of the wrong placement of nodes is diluted by the enlargement of the network size. Furthermore, the growth of the modularity value between the interval [50,100] is most notable while the following increment gradually decreases.

*Effect of average degree on community detection.* Next, we explore the impact of average node degree. We use networks of average degree 2, 3 and 4. We fix the network size to 250 nodes and apply the uniform model to assign interaction probabilities. Figure 6b shows the results. Similar to previous experiment, for all degree settings, our method obtains the highest modularity value. We observe that the average modularity value of all methods decreases notably with the increasing average degree. We conjecture that the reason behind this is that as the number of edges increases, the connectivity between different modules increase as well, thus reducing the modularity value.

*Effect of probability model on community detection.* Finally, we focus on the impact of probability model. For each network topology model, we generate 10 networks with size 250 and average degree 3. For each network, we assign probability values to interactions using three probability models: identical, uniform and normal distribution. We set the probability of all edges to 0.5 in the identical model. Three different normal distribution models are used in our experiment. Three models have the same mean (0.5) but varying standard deviation values (0.1, 0.2 and 0.3). This way, we test a wide spectrum of possible variations in interaction probabilities ranging from no variation (identical model) to very large variation (normal distribution with 0.3 standard deviation). Similar to the previous section, identical probability model is meaningless to apply when threshold method is used for community identification. Threshold method yields the same result as the binary method for small threshold. Because of that we do not report the threshold method for the identical probability model. Figure 6c presents the results.

Our results demonstrate that our method produces the highest modularity value overall across different probability models. Similar to previous experiment, sampling method have similar modularity value with binary method. In the extreme case, when all probabilities are identical, we observe that binary/sampling method achieves slightly better modularity than our method. However, when the edge probability values are heterogeneous, we observe that our method performs best. Furthermore, we observe that as standard deviation increases, the gap between the expected modularity value of our method and the binary/sampling method grows. The modularity value of the threshold method also grows with increasing standard deviation. It however consistently remains below that of our method. The main reason behind it is that all methods we tested except the binary/sampling method are sensitive to the distribution of the edge probabilities. Thus they can adapt to variations in interaction probabilities better.

In summary, our experiments demonstrate that our method is robust and can successfully identify communities of high quality. Both network size and average degree have great effect on our method. Furthermore, our method can adapt to a wide spectrum of edge probability distributions.

Next, we summarize our community detection results on cancer networks.

*Community detection in cancer networks.* In this section, we analyze the similarities and differences among some cancer types with respect to the community structure in the cell cycle pathway using our method. Recall that the interaction probabilities can change across different cancer types. We compare the similarities of community structures for different cancer types using Rand Index (RI). RI is the ratio of the number of pairs of nodes that are placed in the same way in both partitionings to the total number of pairs. Given two partitionings of a network with |*V*| nodes, $\mathcal {C}_{1}$ and $\mathcal {C}_{2}$, the total number of pairs of nodes is $\frac {1}{2}|V|(|V|-1)$. We denote the number of pairs of nodes that are in the same community and different community in both partitionings with *a*_0_ and *a*_1_, respectively. Thus, we calculate the rand index between two partitionings as $RI(\mathcal {C}_{1}, \mathcal {C}_{2})=\frac {2(a_{0}+a_{1})}{|V|(|V|-1)}.$ Figure 4b plots the results.

We observe that the community structures of the cell cycle pathway of all cancer types have substantial similarities. This suggests that the functions of most genes in the cell cycle pathway are consistent across different cancer types. Especially, the community structures of breast and leukemia cancer are highly similar as well as those of prostate and colon cancer. Community structure for lung cancer however shows some deviation from other cancer types.

We conjecture that nodes in the same community tend to serve related functions. To test this conjecture, we do the literature analysis on pairs of nodes in the same community as follows. Given a specific cancer type, for each possible pair of nodes in the network, we count the number of publications in PubMed containing these two genes and the specified cancer. We denote the mean and standard deviation of the number of publications with *μ* and *σ* respectively. For each pair of genes in the same community with *η* publications, we calculate its z-score denoted with *z* as $z=\frac {\eta -\mu }{\sigma }.$ We repeat this process for all cancer types and list pairs of genes whose z-scores are greater than 2. Table 2 presents the results. We observe that the gene pairs our method places in the same community have very high statistical significance in terms of their publication evidences relating them to cancer. We observe that a few gene pairs have substantial evidence across all cancer types (e.g., CDK4 and CDK2 pair). Also some of the publication evidences for some of the gene pairs are unique to certain cancer types. For instance, (RB1, TP53) pair appears for prostate cancer. Indeed, it is shown that in prostate cancer, the lineage plasticity induced by combined loss of RB1 and TP53 confers resistance to antiandrogen therapy [45, 46].

**Table 2:** The pairs of genes in the same community which have z-score values greater than 2

		Prostate		Breast		Lung		Colon		Leukemia	
Interaction		A	B	A	B	A	B	A	B	A	B
CDK4	CDK2	84	14.8	179	11.4	80	9.1	70	12.1	92	8.2
TP53	CDKN1A	26	4.4	82	5.1	54	6.0	48	8.2	52	4.5
CDK1	CDK2	23	3.9	49	3.0			23	3.8	44	3.8
HDAC1	HDAC2	13	2.1			22	2.3	23	3.8	41	3.5
CDK4	CDK6	45	7.8	141	9.0	62	7.0				
CDK6	CDK2	29	4.9	50	3.0	26	2.7				
CDK1	CDC25C	17	2.8			20	2.0				
CDKN1A	MDM2	24	4.0					26	4.3		
TP53	MDM2	23	3.9					29	4.8		
CDK1	CDK4	14	2.3					15	2.4		
CHEK2	ATM			141	9.0			13	2.0		
ATR	ATM			118	7.5			26	4.3		
TP53	RB1	24	4.0								
CDKN1A	CDKN2A	20	3.3								
CDKN1A	CCNB1			44	2.6						
CDKN1A	PCNA			35	2.0						
CHEK1	CHEK2							15	2.4		
TP53	ATM									143	13.0
RB1	CDKN2A									38	3.2
CDKN2A	ABL1									28	2.3
CHEK1	ATR									25	2.0

A = publication count. B = Z-score. The empty entries indicate that the Z-score is below 2

## Discussion and Conclusion

In this paper, we presented a novel method to count the number of shortest paths in probabilistic biological networks. The dependency among multiple paths in probabilistic network makes shortest path counting a challenging problem. We developed a polynomial model to capture such dependency. Our experiments on both synthetic and real networks demonstrate that in comparison to existing methods, our method accurately counts the shortest paths in probabilistic networks. As an important application of our novel shortest path counting method, we use it to identify communities in biological networks. Our experiments show that we could detect communities of high quality in biological networks and identify their key functional characteristics. In future work, it would be interesting to use both the number and the length of shortest paths to characterize the structure of probabilistic biological networks.

## Appendix:

## Community detection problem

We model a partitioning of network *G* into *k* communities with $\mathcal {C}=\{\mathcal {C}_{1}, \mathcal {C}_{2}, \dots, \mathcal {C}_{k}\}$, such that the following three conditions hold:

- $\forall {i},~~i \in \{1,2,\dots, k\},~~\mathcal {C}_{i} \subseteq V$.
- $\forall {i,j},~~i,j \in \{1,2,\dots,k\}~\text {and}~i\neq j,~~\mathcal {C}_{i} \cap \mathcal {C}_{j} = \emptyset $.
- $\cup _{i=1}^{k} {\mathcal {C}_{i}}=V$.

Each node *v*_*i*_ in the partitioning belongs to exactly one community and we denote its membership with *c*_*i*_. That is *c*_*i*_=*j* if *v*_*i*_∈*C*_*j*_. We represent the network *G* with an *n*×*n* adjacency matrix **A**, where each element *A*_*ij*_=1 if there is connection between *v*_*i*_ and *v*_*j*_, otherwise *A*_*ij*_=0. Consider two nodes *v*_*i*_, *v*_*j*_ ∈*V*. We compute the Kronecker delta function *δ*(*c*_*i*_,*c*_*j*_) as 1 if nodes *v*_*i*_ and *v*_*j*_ are in the same community and 0 otherwise. We denote the modularity value of the given partitioning $\mathcal {C}$ with $Q(G,\mathcal {C})$ and calculate it as [39, 47]

11 $$  Q(G,\mathcal{C})=\frac{1}{2m}\sum\limits_{i,j}\left[A_{ij}-\frac{d_{i} d_{j}}{2m} \right]\delta\left(c_{i},c_{j}\right).  $$

Given these definitions, we formally define the community detection problem next.

### **Definition 2**

(DETECTING COMMUNITIES IN PROBABILISTIC NETWORKS). Given a probabilistic network $\mathcal {G}=(\mathcal {V},\mathcal {E},P)$, community detection problem seeks to find a partitioning $\mathcal {C}$ of $\mathcal {G}$with maximum expected modularity value, which is

12 $$ \underset{\mathcal{C}}{\text{argmax}} \left\{\sum\limits_{G \in \mathcal{D}(\mathcal{G})} Q(G, \mathcal{C}) \cdot \mathcal{P}(G|\mathcal{G})\right\}.  $$

Our method adopts Grivan and Newman’s algorithm [39] (the “GN” algorithm), which works for a single deterministic network, to solve the problem for probabilistic networks without losing accuracy of the result.

### Model edge betweenness in probabilistic networks

The GN algorithm finds communities by calculating edge betweenness and iteratively removing edges with highest betweenness. One common way to calculate the betweenness of an edge is to count the number of shortest paths between all node pairs, which contain this edge. As explained in “Counting shortest paths in probabilistic networks” section, however, the probabilistic nature of biological networks makes the existence of the shortest path between a pair of nodes uncertain. Thus, in probabilistic networks, we cannot calculate the edge betweenness as in deterministic networks. A new formulation of the edge betweenness in probabilistic network is needed.

Given a probabilistic network $\mathcal {G}=(\mathcal {V},\mathcal {E},P)$, for each edge *e*_*i*_, we represent the number of shortest paths going along *e*_*i*_ using a random variable *B*_*i*_. We denote *e*_*i*_’s betweenness with *f*(*e*_*i*_) and calculate it as the expected number of shortest paths going along *e*_*i*_ if *e*_*i*_ is present, mathematically,

13 $$ f\left(e_{i}\right)=\frac{\text{Exp}\left(B_{i}\right)}{p_{i}}.  $$

To compute the distribution of *B*_*i*_, one naive way is to count the number of shortest paths for each alternative deterministic network topology of $\mathcal {G}$. Counting shortest paths requires iterating over every pair of nodes. Given a specific deterministic network $G \in \mathcal {D}(\mathcal {G})$, a source node *s* and a sink node *t*, we denote the number of shortest paths between nodes *s* and *t* containing the edge *e*_*i*_ with *N*((*s*∼*t*,*e*_*i*_)|*G*). Notice that *N*(*s*∼*t*,*e*_*i*_|*G*) is not a random variable as the number of shortest paths is certain in deterministic networks.Using these definitions, we compute the expected value of *B*_*i*_ as

14 $$ {\begin{aligned} \text{Exp}\left(B_{i}\right) = \sum\limits_{s,t} Pr\left(B_{s \sim t}>0\right)^{-1} \sum\limits_{G\in \mathcal{D}(\mathcal{G})} N\left(\left(s \sim t, e_{i}\right) | G\right) \cdot \mathcal{P}(G|\mathcal{G}) \end{aligned}}  $$

Equation 14 contains two summations. The inner summation enumerates the distribution of the number of shortest paths connecting nodes *s* and *t* containing *e*_*i*_. We denote this distribution with *B*_*s*∼*t*,*i*_. The outer summation in this equation normalizes this with the inverse of the probability that *s* and *t* are connected through at least one path. That is, for each pair of nodes *s* and *t*, we consider its contribution to the edge betweenness of each edge only if there exists paths connecting *s* and *t*.

Here our goal is to calculate the betweenness value for all edges. The naive solution is to calculate the distribution of *B*_*s*∼*t*,*i*_ for each edge *e*_*i*_. This is however computationally expensive. We observe that for a given pair of nodes *s* and *t*, and two edges *e*_*i*_ and *e*_*j*_ on a path between *s* and *t*, computation of the two values *N*((*s*∼*t*,*e*_*i*_)|*G*) and *N*((*s*∼*t*,*e*_*j*_)|*G*) will contain many common terms. We avoid recomputing these terms by counting the number of shortest paths between *s* and *t* without specifying an edge. We then distribute the value to each edge between those two nodes. Next, we explain how to calculate *B*_*s*∼*t*,*i*_ in detail.

Probability distribution of *B*_*s*∼*t*_ is calculated as explained in “Counting shortest paths in probabilistic networks” section. In fact, we are able to calculate *B*_*s*∼*t*,*i*_ efficiently during the calculation of *B*_*s*∼*t*_. The occurrence of a special term $\alpha _{ij}t_{i}^{j}$ means that there are *j* shortest paths with length *i* in $\mathcal {H}_{s,t}$ connecting a pair of nodes with probability *α*_*ij*_. In the definition of edge betweenness, multiple shortest paths between a pair of nodes are given equal weights summing to 1 [5]. Thus, we assign each such path with a weight of 1/*j*. Assume that *H*_*r*_ is one of the shortest paths. For each edge $e_{q} \in \mathcal {E}(H_{r})$, 1/*j* of all the shortest paths contain *e*_*q*_ with probability *α*_*ij*_. Thus, for every occurring of special terms, we check the *x* variables that lead to the replacing of variable *t*, locate these *x* variables to corresponding paths and append the probability distribution to *B*_*s*∼*t*,*q*_ for each *e*_*q*_ on these paths. For example, consider polynomial terms resulting from $\mathcal {Z}_{a,d}$. For the polynomial term *p*_1_*x*_1_, we replace *x*_1_ with *t*_1_ after applying collapse operator *ϕ*_1_(). Then for each edge on the path *H*_1_, there is one shortest path going through it with probability *p*_1_. Here, the edge is *e*_1_. For another polynomial term $q_{1}p_{2}p_{3}p_{4}p_{5}p_{7}x_{2}^{3} x_{3}^{3} x_{4}^{3} x_{5}^{3}$, after applying collapse operators *ϕ*_2_() and *ϕ*_3_(), we obtain $q_{1}p_{2}p_{3}p_{4}p_{5}p_{7}t_{3}^{2}$ where *x*_2_ and *x*_3_ are replaced with *t*_3_, and *x*_4_ and *x*_5_ are removed as the polynomial term becomes a special polynomial term. For each edge on the path *H*_2_, there is 1/2 shortest path going through it with probability *q*_1_*p*_2_*p*_3_*p*_4_*p*_5_*p*_7_. It is similar for each edge on the path *H*_3_. Notice that for edge *e*_4_ here, there will be 1 (i.e., 1/2 + 1/2) shortest path going through it with probability *q*_1_*p*_2_*p*_3_*p*_4_*p*_5_*p*_7_ as *e*_4_ appears on both paths *H*_2_ and *H*_4_. We repeat this process while calculating the distribution of *B*_*s*∼*t*_ to obtain the distribution of *B*_*s*∼*t*,*i*_. By iterating over each pair of nodes, we obtain the distribution of *B*_*i*_ and calculate Exp(*B*_*i*_).

## Overview of Newman-Grivan’s divisive algorithm

Here, we briefly describe the classical Newman-Grivan’s divisive algorithm [39] for detecting communities in deterministic networks, as our method utilizes the same idea in that study. This work finds communities based on a measure called *edge betweenness*. The betweenness favors edges between communities and disfavors edges within communities. One common way to calculate the betweenness of an edge is to count the number of shortest paths between all node pairs, which contain this edge. More specifically, consider a pair of nodes *s* and *t*. Let us denote the number of shortest paths connecting *s* and *t* with *ν*. Among those paths, let us denote the number of paths which contain the edge under consideration with *ν*^′^. We compute the contribution of the node pair (*s*, *t*) to the betweenness of that edge with *ν*^′^/*ν*. We repeat this for all possible node pairs and report the sum of their contribution as the betweenness of that edge.

Under this definition, briefly, the Newman-Grivan algorithm works iteratively as follows. First, it calculates betweenness values for all edges. Then it removes the edge with the highest betweenness from the given network and recalculates betweenness for the remaining edges. It repeats this process until the graph is empty. It then picks the iteration which yields the highest modularity value and reports the connected components of the network as the community structure of that network. We refer the interested readers to Newman et al. [39] for further details.

### Computing modularity in probabilistic networks

In this section, we discuss how we calculate the modularity value of a partitioning of a probabilistic network in detail. Recall from our problem definition (Definition 2) that given a probabilistic network $\mathcal {G}=(\mathcal {V},\mathcal {E},P)$ and a partitioning $\mathcal {C}$ of $\mathcal {G}$, we calculate its expected modularity value denoted with $\mathcal {Q}$ as

15 $$  \mathcal{Q} = \sum\limits_{G \in \mathcal{D}(\mathcal{G})} Q(G, \mathcal{C}) \cdot \mathcal{P}(G|\mathcal{G}).  $$

Notice that the above formulation enumerates all possible deterministic network topologies. This however is infeasible as the number of deterministic network topologies grows exponentially with the number of edges. To tackle this problem efficiently, we develop a polynomial model to compute the expected modularity value.

Before describing our polynomial model, we first take a look at another equivalent way to calculate the modularity value of a partitioning for a deterministic network. Given a specific deterministic network topology $G \in \mathcal {D}(\mathcal {G})$ and a partitioning $\mathcal {C}$ of *G*, for each community $C_{i} \in \mathcal {C}$, we denote the number of edges within *C*_*i*_ and the sum of the degrees of the nodes in *C*_*i*_ with $l_{C_{i}}$ and $d_{C_{i}}$ respectively. Since the only contributions to $Q(G, \mathcal {C})$ arise from the node pairs belonging to the same community (see Eq. 11), we group these contributions while iterating over each community and calculate $Q(G, \mathcal {C})$ as

16 $$ Q(G, \mathcal{C}) = \sum\limits_{C_{i} \in \mathcal{C}} \left[ \frac{l_{C_{i}}}{m} - \left(\frac{d_{C_{i}}}{2m} \right)^{2} \right].  $$

By injecting the above equation to Eq. 15, we rewrite Eq. 15 as

17 $$  \mathcal{Q} = \sum\limits_{C_{i} \in \mathcal{C}} \sum\limits_{G \in \mathcal{D}(\mathcal{G})} \left[ \frac{l_{C_{i}}}{m} - \left(\frac{d_{C_{i}}}{2m} \right)^{2} \right] \cdot \mathcal{P}(G|\mathcal{G}).  $$

This equivalent way to calculate the expected modularity value summarizes the contribution of each community to expected modularity value.

Now we are ready to define our polynomial model. Consider a probabilistic network $\mathcal {G}=(\mathcal {V},\mathcal {E},P)$ and a partitioning $\mathcal {C}$ of $\mathcal {G}$. For each community $C_{r} \in \mathcal {C}$, we build an *xyz-polynomial* and denote it with $W_{C_{r}}$. To do that, consider the edges into three categories. Those that connect the nodes belonging to *C*_*r*_ constitute the first category. For each edge *e*_*i*_ in this category, we construct an edge polynomial as

$$X_{i}=p_{i}x+q_{i}.$$

This edge polynomial contains two terms. The former one represents that edge *e*_*i*_ belonging to the first category is present with probability *p*_*i*_. The latter one indicates the absence of *e*_*i*_. The edges between community *C*_*r*_ and other communities, fall into the second category. Similarly, for each edge *e*_*i*_ in this category, we construct an edge polynomial as

$$Y_{i}=p_{i}y+q_{i}.$$

The remaining edges make up the third category. We construct an edge polynomial for each edge *e*_*i*_ in this category as:

$$Z_{i}=p_{i}z+q_{i}.$$

After multiplying all these edge polynomials, the xyz-polynomial takes the following form:

$$W_{C_{r}}=\sum \alpha_{ijk}x^{i}y^{j}z^{k}.$$

Notice that each term of this xyz-polynomial, i.e., *α*_*ijk*_*x*^*i*^*y*^*j*^*z*^*k*^, describes a possible deterministic network topology which has *i* edges in *C*_*r*_, *j* edges between *C*_*r*_ and other communities, and *k* remaining edges exists with probability *α*_*ijk*_. Under this specific deterministic topology, the number of edges within *C*_*r*_, $l_{C_{r}} = i$; the sum of degrees of nodes within *C*_*r*_, $d_{C_{r}} = 2i+j$ (each edge in the first category contributes two degrees while each edge in the second category contributes one degree); the number of edges, *m*=*i*+*j*+*k*. Thus, we compute the modularity value of *C*_*r*_ as

$$\mathcal{Q}_{C_{r}}=\sum\limits_{i,j,k} \alpha_{ijk} \left[ \frac{i}{i+j+k} - \left(\frac{2i+j}{2(i+j+k)} \right)^{2} \right].$$

By repeating this process for each community and adding the contribution of each community up, we obtain the expected modularity value. It is worth noting that, computing the three polynomials *X*_*r*_, *Y*_*r*_, and *Z*_*r*_ is trivial since each one contains only one variable. More specifically, let us denote the number of edges in a category with *κ*. Computing the above polynomial has *O*(*κ*^2^) time complexity if we iteratively multiply one polynomial at a time.

## Effect of the threshold value

**Fig. 7:**
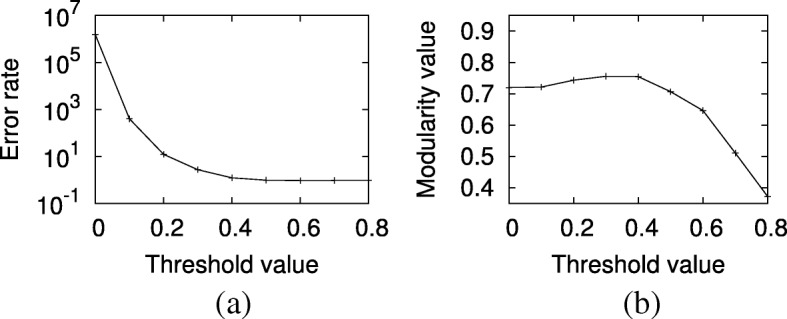
Relative error rate (**a**) and expected modularity value (**b**) of threshold method on synthetic networks

Recall that the threshold method maintains the set of edges with probability value above a given threshold value and removes the remaining edges. Thus, the outcome of this method depends on the threshold value. In this section, we test the performance of the threshold method for varying values of threshold on both the shortest path counting and the community detection problem. We run our experiment on synthetic networks of various sizes; 50, 100 and 250. In particular, we generate 10 random LFR benchmark networks with average degree three for each network size. We assign the probability value using uniform model. For the threshold method, we vary the threshold value from 0 to 0.8 at increments of 0.1. For each threshold value setting, we run experiment on all generated networks, and report the relative error of the shortest path counting method and average modularity value. We also run our method on the same set of networks and report the average modularity. Figure 7 plots the results.

We observe that the error rate of threshold method first goes down sharply with the increasing threshold value (Fig. 7a). It then becomes flat after value 0.4. The major reason is that smaller threshold values keep most edges of the network which makes the performance of the threshold method much like that of the binary method. The larger threshold values however remove most edges of the network which makes the number of shortest paths between most pairs of nodes much close to 0. As a result, the error rate is close to 1. In our shortest path counting experiments we set the threshold value to 0.6. In Fig. 7b, we observe that our method achieves higher expected modularity value than the threshold method regardless of the threshold value. The modularity value of the threshold method first grows with the increasing threshold value. It then falls sharply. It obtains the peak value when the threshold is 0.3. This is possibly because too small/large threshold leads to the retaining/removing most of the edges of the network. Either case may make the threshold method underutilize the information available in the interaction probabilities. As a result, a suitable threshold value is necessary for the threshold method. However, the varying distribution of edge probabilities of different probabilistic networks makes it difficult to set a fixed threshold value for the threshold method. In our community detection experiments, we fix the threshold value of the threshold method to 0.3 as it obtains the best value on the average across a broad spectrum of parameter settings.

## Shortest path counting utilizes a nonlinear function

In this section, we show that shortest path counting method uses a nonlinear function. First, we define the key terms that are essential to describe our method. We then show why the function used in our method is nonlinear.

Let $\mathcal {G}=(\mathcal {V},\mathcal {E})$ denote a probabilistic network where $\mathcal {V}$ and $\mathcal {E}$ represent the node and edge sets, respectively. Suppose that *u* and *v* are two nodes in the network $\mathcal {G}$, and *G*_*j*_ is the *j*th possible deterministic instantiation of the probabilistic network $\mathcal {G}$. We denote the *i*th path between the nodes *u* and *v*, and its length with *x*_*i*_ and *L*_*i*_, respectively. Let *Y*_*ij*_ be a Boolean variable that represents whether the path *x*_*i*_ exists between nodes *u* and *v* in *G*_*j*_. We formally define *Y*_*ij*_ as

$$Y_{ij} = \begin{cases} 1 & \text{if}\ x_{i} \in G_{j} \\ 0 & \text{else} \\ \end{cases} $$

Let *Z*_*ijk*_ denote a Boolean variable that compares the path length of *x*_*i*_ (*L*_*i*_) to all other paths in the deterministic network *G*_*j*_. The function *Z*_*ijk*_ is equal to 1 if paths *x*_*i*_ and *x*_*k*_ exist in *G*_*j*_, and length of path *x*_*i*_ (*L*_*i*_) is smaller than the length of path *x*_*k*_ (*L*_*k*_). We formally define *Z*_*ijk*_ as

$$Z_{ijk} = \begin{cases} 1 & \text{if}\ x_{i} \in G_{j}, x_{k} \in G_{j}, \text{and }L_{i} \leq L_{k} \\ 0 & \text{else} \\ \end{cases} $$

Using the Boolean variables *Y*_*kj*_ and *Z*_*ijk*_ we define the shortest path counting function *NSP* (Number of Shortest Paths) as

$$NSP = \sum\limits_{i, j} \left[Y_{ij} \times \prod_{k, i}\left(Y_{kj} \times Z_{ijk}+\left(1-Y_{kj}\right)\right)\right]$$

Please note that given *Y*_*ij*_=1 (i.e., *x*_*i*_ path exists in *G*_*j*_), the inner term *NSP* function ($Y_{ij} \times \prod _{k,i}\left (Y_{kj} \times Z_{ijk}+\left (1-Y_{kj}\right)\right)$) will be zero if *Y*_*kj*_=1 and *Z*_*ijk*_=0 for at least one *k* (i.e., *x*_*i*_ and *x*_*k*_ paths exist in *G*_*j*_, and path length of *x*_*i*_ is longer than path length of *x*_*k*_). The sum of all possible products in *NSP* function gives the number of shortest paths in the network $\mathcal {G}$. *NSP* is a nonlinear function due to the products involved in its definition.

## Abbreviations

GN: Grivan and Newman
KEGG: Kyoto encyclopedia of genes and genomes
LFR: Lancichinetti-Fortunato-Radicchi
MINT: The molecular interaction database
NCBI: The national center for biotechnology information
RI: Rand index
STRING: Search tool for the retrieval of interacting genes/proteins
TCGA: The cancer genome atlas
